# Influenza surveillance and vaccine policy in Thailand—a historical perspective

**DOI:** 10.1016/j.lansea.2025.100663

**Published:** 2025-09-10

**Authors:** Martha P. Montgomery, Prabda Praphasiri, Darunee Ditsungnoen, Pasakorn Akarasewi, Malinee Chittaganpitch, Pilaipan Puthavathana, Khanchit Limpakarnjanarat, Ponthip Wirachwong, Tawee Chotpitayasunondh, Narumol Sawanpanyalert, Chaninan Sonthichai, William W. Davis, Sonja J. Olsen, Supamit Chunsuttiwat

**Affiliations:** a Thailand Ministry of Public Health–U.S. Centers for Disease Control and Prevention Collaboration, Nonthaburi, Thailand; b U.S. Centers for Disease Control and Prevention, Atlanta, GA, USA; c Department of Disease Control, Ministry of Public Health, Nonthaburi, Thailand; d National Institute of Health, Department of Medical Sciences, Ministry of Public Health, Nonthaburi, Thailand; e Mahidol University, Bangkok, Thailand; f Government Pharmaceutical Organization, Bangkok, Thailand; g Queen Sirikit National Institute for Child Health, Bangkok, Thailand; h Department of Medical Services, Ministry of Public Health, Nonthaburi, Thailand

**Keywords:** Influenza, Vaccination, Thailand, Surveillance

## Abstract

Prior to 2000, influenza burden in Thailand and other low- and middle-income countries was underappreciated, and influenza vaccination was uncommon. For the last two decades, Thailand Ministry of Public Health (MOPH) and U.S. Centers for Disease Control and Prevention have collaborated to understand influenza burden and the costs and benefits of influenza vaccination in Thailand. Built on a long-standing national disease notification system, Thailand MOPH established robust surveillance platforms for pneumonia and influenza, which provided insights into seasonality, disease incidence, and populations at risk for severe disease. In 2004, human cases of avian influenza brought attention to influenza's pandemic potential. Concern for an influenza pandemic combined with evidence of the cost effectiveness of influenza vaccination accelerated vaccine policy. Surveillance and vaccination policy were leveraged for and strengthened by the 2009 influenza H1N1 and COVID-19 pandemics. This personal view documents Thailand's experience in developing influenza surveillance and influenza vaccination policy.

**Search strategy and selection criteria**

References for this Review were identified through the authors' selected works and by searches of PubMed for the terms “Thailand” and “influenza” with no restrictions on publication years or language. References within the identified articles were further reviewed to identify additional references. The total number of references reviewed was not tracked systematically.

## Introduction

In the early 2000s, the burden of influenza in Thailand relative to other public health problems was largely unknown and underappreciated, and less than 1% of people were vaccinated against influenza annually. For the last two decades, Thailand Ministry of Public Health (MOPH) and U.S. Centers for Disease Control and Prevention have collaborated to gather evidence on influenza burden and the costs and benefits of vaccination. In Thailand, the momentum for influenza vaccine policy was accelerated by events like the 2004–2006 avian influenza outbreaks and the 2009 H1N1 influenza pandemic. These events, along with improved understanding of influenza disease burden and vaccine cost effectiveness, provided justification to policy makers to make long lasting investments in influenza prevention, including domestic vaccine manufacturing and vaccination programs. Presently, Thailand recommends seasonal influenza vaccine for all groups recommended by the World Health Organization (WHO) Strategic Advisory Group of Experts on Immunization, which includes pregnant women, older adults (65 years and older), people with comorbidities, and health workers, plus additional groups in Thailand like poultry cullers, young children, people with mental disabilities, and people living or working in prisons.^1^

Vaccine policies in Thailand fall under the oversight of multiple bodies, including the National Vaccine Committee and its subcommittees. One subcommittee is the Thailand Advisory Committee on Immunization Practices (ACIP), which makes recommendations on vaccination timing and target groups.^2^ After the Thailand ACIP makes its recommendations, the Committee on National List of Essential Vaccines considers the vaccine for inclusion in the National List of Essential Vaccines. If included in the National List of Essential Vaccines, the vaccine is entitled for budget allocation under the Universal Health Coverage scheme, which is managed by the National Health Security Office (NHSO).^2^

Experts in Thailand's MOPH were considering influenza vaccination policy as early as the 1990s,^3^^,^^4^ but three main concerns needed to be addressed to advance vaccine policy. First, cost of influenza vaccine was prohibitively expensive without convincing cost effectiveness data. Secondly, there were technical questions about the optimal timing of annual vaccination and which groups would benefit the most. Third, there was a common perception among the public and professionals that seasonal influenza was not severe.^5^ Influenza surveillance provides critical information to understand influenza burden, seasonal trends, and risk groups, and it can inform public health intervention measures like vaccination strategies. The following sections describe how influenza surveillance helped to address these concerns and how real-world events accelerated influenza vaccination policy decisions in Thailand.

## Respiratory disease surveillance in Thailand 1970–2000

Thailand's MOPH has a well-established National Disease Surveillance Reporting System (R506) dating back to the 1970s.^6^ For the first three decades, case surveillance for influenza and pneumonia consisted of passive reporting from hospital clinicians, which relied on clinician awareness to diagnose and report cases. Influenza cases were diagnosed clinically because laboratory testing for influenza was uncommon.^6^, ^7^, ^8^ Because of the passive nature of surveillance, influenza cases were underestimated.^5^^,^^9^^,^^10^

Around the same time that passive syndromic disease surveillance was established, virologic surveillance for influenza viruses began separately. In 1972, Thailand's National Institute of Health was recognized as a National Influenza Center (NIC), part of a global network of laboratories for influenza surveillance established by WHO in 1952.^11^^,^^12^ The objective of virologic surveillance for the NIC during this period was to support WHO in vaccine strain selection.^11^, ^12^, ^13^ Specimens were collected from a single hospital site, usually in Bangkok or Chiang Mai, and epidemiologic data were limited to age, sex, and date of specimen collection. An influenza virus identified by virologic surveillance in Thailand, influenza A/Bangkok/79 (H3N2), was included in the recommended vaccine formulation in the United States and Northern hemisphere in 1980–1981, 1981–1982, and 1982–1983 seasons.^14^, ^15^, ^16^ To improve the geographic representativeness of virologic surveillance, the NIC added four sites in 2001 along borders with neighboring countries.^12^

Both the passive syndromic surveillance and virologic surveillance systems provided information on influenza seasonality. By the 1980s, it was apparent that influenza in Thailand occurred throughout the year and increased from around June through October with an occasional secondary peak around January and February.^3^^,^^7^^,^^13^^,^^17^ Disease surveillance was effective for monitoring the frequency of clinical respiratory syndromes but lacked the laboratory testing capacity to provide pathogen-specific surveillance. Virologic surveillance provided valuable information on influenza virology but could not describe the frequency of infections or identify groups at risk for severe disease. Disease surveillance and virologic surveillance for respiratory infections operated in parallel, with regular information exchange and discussion, until the early 2000s.

## Launch of active pneumonia surveillance in 2001

In 2001, the U.S. CDC started the International Emerging Infections Program.^8^ In collaboration with Thailand MOPH, the first site was launched in Thailand with a focus on conducting active surveillance for pneumonia. Globally, respiratory infections have long been recognized as a leading cause of mortality, but by the 1990s the most common etiologic agents and effective case management strategies in low- and middle-income countries were unknown.^18^, ^19^, ^20^ By the early 2000s, improved access to vaccines and treatments for *Haemophilus influenzae*, *Streptococcus pneumoniae*, and influenza in low- and middle-income countries drove an interest to understand the relative contribution of these pathogens to morbidity and mortality.^8^^,^^21^ Thailand's passive National Disease Surveillance Reporting System included pneumonia but did not include pathogen-specific information. Thailand had conducted studies in the 1980s to understand the etiologic agents of acute respiratory infection in children as part of a multinational research effort.^22^, ^23^, ^24^ Active pneumonia surveillance supported by the CDC International Emerging Infections Program expanded on this knowledge in key ways. The active pneumonia surveillance system used a population-based design, which allowed for the calculation of incidence and burden estimates, and the addition of laboratory testing capacity allowed for surveillance of specific pathogens.^6^^,^^8^

In Thailand, Sa Kaeo province was selected as the first active pneumonia surveillance site in 2002, and Nakhon Phanom province was added one year later.^8^ Laboratory training and reagents were provided to strengthen laboratory capacity for specimen collection, transportation, and pathogen isolation.^8^ Because transporting and testing viral specimens was easier than bacterial specimens, viral respiratory pathogens like influenza viruses, parainfluenza viruses, respiratory syncytial viruses (RSV), respiratory adenoviruses, and human metapneumovirus were the first pathogens studied.^8^^,^^25^, ^26^, ^27^, ^28^

Active pneumonia surveillance provided a foundation for understanding the burden of influenza on communities and on public health and medical systems in Thailand. Analyses of active surveillance data from 2003 to 2004 showed that outpatient influenza cases were up to 43 times greater than estimated previously from passive surveillance.^9^ Nationally, influenza pneumonia was estimated to be associated with up to 75,000 hospitalizations and nearly 1 million outpatient visits annually during 2003–2004.^9^ This was believed to be an underestimate of all influenza hospitalizations because many influenza virus infections do not present with pneumonia and were not included in the study. During 2005–2008, influenza was responsible for an estimated 10% of pneumonia hospitalizations.^5^ In the community, as many as 6% of people experienced influenza acute respiratory infections annually.^10^^,^^29^^,^^30^

Along with documenting the frequency and seasonality of influenza cases, surveillance data from 2003 to 2004 were combined with Thailand national databases, community surveys, medical records, and patient interviews to estimate direct and indirect costs of influenza.^9^ During September 2003 through August 2004, the estimated costs of hospital admissions and outpatient visits for influenza was between $23 million and $63 million US dollars in total losses to Thailand.^9^ Just over half of the estimated costs were attributed to lost productivity, while 43% was attributed to direct medical costs.^9^ Outpatient visits for influenza were estimated to result in over 3 million days of lost work and 1.7 million missed days of school.^9^ A household survey in 2003 found that the average cost of an episode of influenza could cost 20% of the average monthly household income, and nearly half of households experiencing an episode of influenza-like illness reported taking out a personal loan to cover medical expenses.^10^

## Human cases of avian influenza in Thailand lead to dedicated influenza surveillance in 2004

Shortly after the active pneumonia surveillance platform was established, in late December 2003, a young boy in Thailand developed an acute upper respiratory tract infection that progressed to fatal pneumonia.^31^ This was one of the first confirmed human cases of avian influenza A(H5N1) virus infection in Thailand. The virus was detected on January 23, 2004, and reported the same day. From 2004 through 2006, Thailand experienced four waves of human avian influenza virus infections and detected 25 cases, including 17 deaths.^21^^,^^32^ To interrupt transmission, over 30 million poultry, mostly chickens, were culled with compensation provided to poultry owners.^33^ Human avian influenza cases were reported contemporaneously from nearby Viet Nam, Cambodia, and Indonesia.^34^ Thailand provided epidemiologic investigation and laboratory assistance to neighboring Lao People's Democratic Republic when the first human case of A(H5N1) was detected in 2007.^35^ The detection of avian influenza with high mortality in the region raised concern among the public and public health leaders about the pandemic potential of influenza.^32^ In 2004, health care personnel and poultry cullers were vaccinated against seasonal influenza, and in 2005, the Thailand ACIP issued an ongoing recommendation for seasonal influenza vaccine for these two groups.^36^ Although seasonal influenza vaccine would not protect against influenza A(H5N1), Thailand public health leaders were concerned about theoretical reassortment of human seasonal and avian influenza viruses and wanted to strengthen preparedness for a possible influenza pandemic.^32^^,^^37^

The concern for avian influenza among global leaders was evident during the 56th World Health Assembly in May 2003, when WHO urged member states, “to draw up and implement national plans for preparedness for influenza pandemics”.^38^ Thailand began plans on influenza pandemic preparedness, and on January 25th, 2005, Thailand endorsed its first National Strategic Plan for Influenza Pandemic Preparedness, 2005–2007 (hereafter “National Strategic Plan”).^37^ The National Strategic Plan outlined Thailand's strategy to strengthen influenza surveillance as one component of its larger pandemic preparedness strategy. Thus, in addition to leveraging the existing pneumonia surveillance, Thailand's MOPH added dedicated influenza surveillance in 2004.^7^^,^^32^ Instead of a population-based design with reporting from all hospitals in a province, influenza surveillance enrolled one or two sentinel hospitals per province from multiple provinces to provide wider geographic representation. Using sentinel surveillance in more provinces rather than population-based surveillance would hopefully provide earlier detection of large influenza outbreaks while remaining within the constraints of available resources. Influenza surveillance applied standardized case definitions and focused initially on influenza-like illness in outpatient clinics. Surveillance of hospitalized patients was added in 2010.^32^ Sentinel surveillance monitors a representative population subset and is not intended to identify all cases of emerging respiratory threats of pandemic potential. Therefore, MOPH also established event-based reporting of respiratory clusters to address pandemic early warning needs, reviewed autopsy data from pneumonia deaths, and collected data from passive reporting systems.^39^

The establishment of influenza sentinel surveillance was also accompanied by enhancements in epidemiologic, clinical, and laboratory capacity. Thailand's MOPH developed clinical practice guidelines for the treatment and prevention of influenza. They also developed trainings for health care providers on diagnosis and management of influenza and other emerging pathogens. The Virology Association of Thailand was instrumental in conducting workshops, lectures, and demonstrations to train new generations of public health laboratorians to support surveillance of influenza and other new and emerging respiratory pathogens. The Influenza Study Foundation of Thailand, a voluntary group of professionals established in response to avian influenza, has actively provided technical updates on influenza for medical staff countrywide and has promoted influenza research over the past two decades.

## Pandemic concern drives vaccine manufacturing capacity and vaccination programming, 2004–2009

In addition to strengthening influenza surveillance, the National Strategic Plan outlined a strategy to develop domestic influenza vaccine manufacturing capacity.^40^^,^^41^ Leadership in Thailand recognized the importance of self-reliance in vaccine manufacturing in the context of a pandemic, stating, “if an influenza pandemic outbreak takes place in the near future, only those countries which would have the capacity to manufacture vaccines and antiviral drugs would be able to make use of the products”.^37^ In 2007, MOPH requested government support for a pandemic influenza preparedness proposal consisting of two major initiatives—local influenza vaccine manufacturing and influenza vaccination for groups at higher risk of influenza virus exposure or for complications from influenza. Although the proposal implied considerable and continuous investments, it was finally approved in the same year. Vaccine manufacturing was tasked to the Government Pharmaceutical Organization while influenza vaccination programming was assigned to the Department of Disease Control.

WHO also recognized that global vaccine manufacturing would be insufficient to produce an adequate number of vaccine doses in the context of a pandemic.^40^ In 2006, WHO developed the Global Action Plan for Influenza Vaccines to increase global manufacturing capacity, in part by expanding manufacturing capability in developing countries.^40^ In 2007, as a partner of WHO's Global Action Plan, Thailand received support from U.S. Biomedical Advanced Research and Development Authority to increase influenza vaccine manufacturing capacity.^41^ In the first three years, Thailand achieved numerous milestones, including constructing and renovating manufacturing facilities; passing WHO and Thailand FDA inspections; achieving compliance with current Good Manufacturing Practices; developing specialized laboratory skills; completing animal, phase I, and phase II clinical trials; and strengthening partnerships with internal, external, and academic partners. Following successful demonstration of safety and immunogenicity, an H5N2 live attenuated influenza vaccine was licensed by Thai FDA for pandemic use under Emergency Use Authorization.^42^

The National Strategic Plan aimed to increase vaccine demand by expanding seasonal influenza vaccine recommendations to new groups. Although the campaign to establish influenza vaccine manufacturing capacity in Thailand and other Global Action Plan counties was driven mainly by the need to ensure vaccine access in the face of a pandemic, it was well recognized that once the manufacturing capacity for pandemic (monovalent) vaccine is established, it must be maintained by production of seasonal (multivalent) influenza vaccine during inter-pandemic periods. As the National Strategic Plan stated, “more use of influenza vaccines should be encouraged [to] allow the country to immediately expand the vaccination services, particularly during pandemic outbreaks”.^33^

Surveillance data helped to identify the groups with increased risk for influenza virus infection or severe influenza that would most benefit from vaccination. Analyses from active pneumonia surveillance showed that young children and older adults were most impacted by influenza^5^ and that young children, older adults, and people with chronic medical conditions had an increased risk of hospitalization for influenza pneumonia compared with the general population.^43^ Results from a randomized controlled vaccine trial during 2003–2004 showed that influenza vaccination for patients with chronic obstructive pulmonary disease (COPD) was cost saving.^44^ The authors concluded that, “influenza vaccination should be recommended to all patients with COPD with the higher priority provided to the patients with more severe COPD.”^44^ Another randomized controlled vaccine trial conducted among community-dwelling, older adults in 1998–1999 demonstrated effectiveness of influenza vaccination in producing a serologic response and reducing influenza-like illness but was inconclusive on the economic benefits.^45^ Nevertheless, the authors recommended vaccination for older adults in anticipation of a severe epidemic.^45^ In 2008, the Thailand ACIP recommended influenza vaccine for adults aged 65 years and older with chronic medical conditions, followed by an extension in 2009 to people of all ages with chronic medical conditions.^46^ NHSO purchased vaccine and provided influenza vaccination for free to these groups through the Universal Health Coverage scheme.^41^

## Impact of 2009 H1N1 and COVID-19 pandemics

In March 2009, the first cases of pandemic influenza A H1N1 were detected in Mexico.^47^ By early May, the first imported case in Thailand was reported, and within two months, the influenza A(H1N1)pdm09 virus had spread throughout the country.^42^ During the 2009 H1N1 pandemic response, the population-based pneumonia surveillance system was leveraged to estimate the burden of the influenza pandemic on hospitalizations in Thailand.^48^ Influenza hospitalization rates during 2009–2010 were highest in young children and older adults, and rates were higher than what was being reported from western countries.^48^ Even prior to the 2009 H1N1 pandemic, evidence for vaccinating children against seasonal influenza had been accumulating. A hospital-based study in Bangkok during 2004–2005 showed that influenza was an important contributor to lower respiratory tract infections in children.^49^ Modeling of pneumonia surveillance data from 2005 to 2008 estimated that almost one-third of all pediatric influenza pneumonia hospitalizations in Thailand (ages 0–17 years) could be prevented by vaccinating 85% of children aged 6 months through 4 years.^50^ A modeling study using influenza sentinel surveillance data from 2005 to 2009 compared the cost effectiveness of different vaccine formulations (inactivated and live-attenuated) and age groups (preschool, primary, and secondary school), and concluded that influenza vaccination for school-aged children in Thailand would be cost effective.^51^ The 2009 influenza pandemic accelerated the Thailand ACIP to recommend seasonal influenza vaccine for children aged 6–35 months in 2010. Recommendations were also added for pregnant women and people with obesity, mental disability, thalassemia, and immune compromise.^36^ NHSO expanded the health benefits under Universal Health Coverage to cover seasonal influenza vaccination for these groups in 2014.

As during the avian influenza outbreaks, the 2009 H1N1 pandemic provided an impetus to strengthen vaccine manufacturing capacity. Through WHO's Global Action Plan for Influenza Vaccines, Thailand received master seed for 2009 H1N1 pandemic strain from Russia. Thailand filled the first lot of live attenuated vaccines by August 2009, completed vaccine clinical trials, and licensed the vaccine for pandemic use, although it was not available in time for widespread use.^42^^,^^52^ Eventually, Thailand purchased 2 million doses of monovalent pandemic H1N1 vaccine and administered the vaccine during January to June 2010.^53^^,^^54^ This was followed by administration of 2 million doses of trivalent vaccine starting in July 2010.^54^

As described in a review by Ungchusak et al., soon after the initiation of the pandemic influenza vaccination campaign in 2009, media reported fetal deaths in pregnant women who were vaccinated against influenza.^53^ These reports might have negatively impacted public perception of vaccine safety for pregnant women. Influenza vaccination for pregnant women declined in Thailand, and vaccine doses were administered to volunteers and soldiers to avoid wastage. The authors of this review emphasized the importance of risk communication.^53^ Studies have supported the safety of maternal influenza vaccination, including influenza vaccines used during the 2009 influenza pandemic, and the lack of association between 2009 H1N1 pandemic influenza vaccination and fetal deaths.^55^

This negative perception might have had a long-lasting impact on seasonal influenza vaccination campaigns in Thailand. In 2012, three years after the initial influenza vaccine recommendation for pregnant women, fewer than 1% of pregnant women in Thailand received the seasonal influenza vaccine.^56^ In 2012–2013, Thailand MOPH surveyed clinicians and pregnant women to assess knowledge, attitudes, and practices related to seasonal influenza vaccination.^57^^,^^58^ These studies helped to refine vaccination program implementation. For example, documentation of influenza vaccination was added prominently to the pink book, a book used by all pregnant women to track maternal and child health visits before and after delivery. In 2015, a fatal case of influenza in an unvaccinated pregnant woman raised awareness of the importance of influenza vaccination during pregnancy.^56^ Despite these efforts, fewer than 10% of pregnant women in Thailand were vaccinated against influenza in 2024.^59^

During the COVID-19 pandemic, Thailand similarly leveraged its influenza vaccine manufacturing experience and infrastructure to conduct clinical trials, license, and manufacture COVID-19 vaccines domestically.^60^ In August 2021, Thailand's National Regulatory Authority for vaccines reached maturity level 3, the second highest WHO classification level for national regulatory systems.^61^ The influenza surveillance system was leveraged for COVID-19 surveillance by adding a multiplex assay that included influenza, SARS-CoV-2, and other respiratory pathogens at sentinel sites. In the early emergency phase of the COVID-19 pandemic, Thailand scaled up respiratory surveillance in multiple settings including at ports of entry (airports, seaports, ground crossings) and in the community in addition to traditional hospital and clinic settings.^62^ However, these surveillance systems were not necessarily sustainable or necessary during interpandemic periods. Leveraging the existing influenza sentinel surveillance system for COVID-19 by using a shared screening case definition and multiplex assay for laboratory confirmation allowed for COVID-19 surveillance to continue sustainably beyond the emergency phase.

## Surveillance continues to inform vaccine policy

Thailand's MOPH continues to conduct surveillance for respiratory illnesses, including acute respiratory infections associated with influenza viruses, SARS-CoV-2, RSV, and other pathogens. Surveillance in Thailand uses complementary approaches of indicator-based (e.g., sentinel) and event-based surveillance across the country. Thailand's NIC routinely submits specimens and viral isolates to WHO Collaborating Centers for global vaccine strain selection. Influenza specimens submitted from Thailand have been included in previously recommended compositions of influenza virus vaccines, including influenza B/Phuket/3073/2013 (B/Yamagata lineage)-like virus and influenza A/Thailand/8/2022 (H3N2)-like virus. In 2022, U.S. CDC provided training to the NIC on influenza sequencing and bioinformatics. Thailand's NIC sequences influenza viruses collected through surveillance to compare circulating viral strains with vaccine strains and assess vaccine match. Thailand's NIC now provides regional assistance in respiratory virus sequencing to other countries.

After recommending influenza vaccine for several groups, Thailand MOPH conducted studies to document influenza vaccine effectiveness, including among adults aged 50 years and older,^46^ people with chronic medical conditions,^63^ young children,^64^ and other risk groups.^65^ Thailand also shares vaccine effectiveness estimates with WHO's Global Influenza Vaccine Effectiveness Collaboration, which are used to inform northern and southern hemisphere influenza vaccine composition.^66^ Changes in influenza vaccine composition and effectiveness from year to year illustrates the need for ongoing monitoring and reanalysis.^64^

Ongoing surveillance and complementary special studies are used to strengthen and refine vaccination programming and clinical practice guidelines. Seasonality of influenza had already been described in early disease surveillance, but analysis of surveillance data from 2000s helped to refine vaccination program timing. In 2008, vaccinations were provided in April and May.^5^ In 2010, influenza vaccinations were delayed to July through October because of the 2009 H1N1 pandemic, which coincided with peak influenza virus activity that year.^46^ In 2011, based on analysis of surveillance data, March and April were proposed as optimal months for influenza vaccination.^32^ This was revised again in response to further analyses; vaccinations are now offered annually during May through August, approximately 1–2 months in advance of the expected increase in cases.^67^

Thailand's influenza vaccine recommendations continue to evolve as new data emerge. Thailand's MOPH responded to several influenza outbreaks in prisons that were detected through event-based surveillance.^68^, ^69^, ^70^ Outbreak investigations described risk factors for rapid influenza transmission in these settings, including limited space for isolation of symptomatic individuals, close sleeping and working spaces, and shared drinking glasses or cigarettes. One study reported that nearly half of all prisons experienced an influenza outbreak over a 3-year period.^69^ A modeling study found that influenza vaccination could be a cost effective measure to prevent outbreaks in prisons.^71^ In 2021, influenza vaccination was recommended for people living or working in prisons as a pilot project, and a recommendation was formalized in 2024.

In 2023, with supportive evidence from surveillance data and epidemiologic cohort studies, Thailand ACIP expanded the influenza vaccine recommendation for young children from 35 months to 60 months. Modeling studies from a prospective cohort of children during 2013 and 2014 showed that vaccinating children against influenza was cost effective, and that expanding vaccine recommendation among children up to age 5 years with coverage of 50% could prevent 121,000 medical visits annually in this age group.^72^^,^^73^ The same study found that hospitalization for an influenza-associated acute respiratory illness could cost 28–42% of a family's median monthly household income.^29^

## Remaining challenges with vaccination coverage

Despite progress in establishing vaccine recommendations, the number of influenza vaccine doses covered by the Universal Health Coverage scheme is insufficient to cover all Thailand ACIP recommended groups due to the high cost of vaccine and limited budget.^74^ As a result, influenza vaccination coverage remains low for several recommended groups, despite generally high acceptance of influenza vaccination.^36^^,^^74^^,^^75^ Influenza vaccination campaigns are organized annually by the Department of Disease Control and NHSO and are conducted from May through August.^59^ Influenza vaccine doses are allocated for target groups and administered doses are monitored to improve service delivery. Influenza vaccine doses are distributed on a first come, first served basis, and differences in vaccination coverage by recommended group are apparent. In 2012, around one in five adults aged 65 years and older were vaccinated for influenza. For children younger than 3 years, fewer than 2% were vaccinated.^36^ From 2013 through 2023, vaccination coverage using publicly procured vaccines among all eligible individuals ranged from 17 to 34%.^76^ In 2024 data from Thailand's Expanded Program on Immunization, coverage is improved for some groups but remains low. Vaccination coverage with publicly purchased doses is highest among health care personnel (85%), lower for older adults (47%) and people with chronic medical conditions (27%), and lowest for pregnant women (6%) and young children (2%).^59^^,^^76^ Providing clear, consistent messaging to patients and health care providers on who is eligible for vaccination and how to access vaccination in Thailand could be helpful, but the key constraint is vaccine supply. In contrast with vaccines that produce lifelong immunity, the need to revaccinate against influenza annually is an important consideration in sustaining influenza vaccination programs.

## Conclusion

Thailand was the first country in the Southeast Asia region to offer publicly funded, seasonal influenza vaccination to select populations.^77^ Thailand's incremental approach to introducing seasonal influenza vaccination starting with health care workers and expanding slowly based on disease risk has been cited as a model for neighboring countries.^77^ In Thailand, development of influenza surveillance and vaccine policy reflects decades of persistence and leadership commitment from MOPH and collaboration with international partners. Change came about not from single studies but from the accumulation of evidence collected systematically as well as real-world events that were important drivers of change.

Establishing dedicated influenza surveillance systems in Thailand helped leadership to address key questions about vaccine policy. Namely, surveillance data provided answers about the burden of disease on the population, the cost of illness to the country, the population groups with most severe outcomes and at highest risk of exposure, the optimal timing of vaccine administration, and the cost effectiveness of vaccination strategies. Vaccination programs in middle income countries, which have competing health priorities and operate with limited budgets, are not prioritized for support from international donors. Influenza vaccination is particularly challenging because of the need to revaccinate annually. Therefore, data on disease burden and cost effectiveness of influenza vaccination provide critical information for Thailand to decide how to prioritize influenza programs relative to other health concerns.

## Contributors

MPM contributed to data curation, formal analysis, writing original draft, and writing – reviewing and editing. PP contributed to formal analysis and writing – reviewing and editing. DD contributed to formal analysis and writing – reviewing and editing. PA contributed to formal analysis and writing – reviewing and editing. MC contributed to formal analysis and writing – reviewing and editing. PP contributed to formal analysis and writing – reviewing and editing. KL contributed to formal analysis and writing – reviewing and editing. PW contributed to formal analysis and writing – reviewing and editing. TC contributed to formal analysis and writing – reviewing and editing. NS contributed to formal analysis and writing – reviewing and editing. CS contributed to formal analysis and writing – reviewing and editing. WWD contributed to formal analysis, supervision, and writing – reviewing and editing. SJO contributed to conceptualization, formal analysis, supervision, and writing – reviewing and editing. SC contributed to conceptualization, formal analysis, supervision, and writing – reviewing and editing.

## Declaration of interests

The authors declare no competing interests.

## Appendix A Supplementary data

Translated Summary in Thai
