# Supplementary material for: Influenza surveillance and vaccine policy in Thailand—a historical perspective
Source: Lancet Reg Health Southeast Asia. 2025 Sep 10;41:100663. doi: 10.1016/j.lansea.2025.100663 (PMC12862145; doi:10.1016/j.lansea.2025.100663)
Supplement: Translated Summary in Thai [file mmc1.docx]

This translation in Thai was submitted by the authors and we reproduce it as supplied. It has not been peer-reviewed. Our editorial processes have only been applied to the original abstract in English, which should serve as reference for this manuscript

ก่อนปี พ.ศ. 2543 การประเมินภาระโรคไข้หวัดใหญ่ในประเทศไทยและประเทศที่มีรายได้ต่ำและปานกลางอื่นๆ มีอยู่อย่างจำกัดและการฉีดวัคซีนไข้หวัดใหญ่ยังไม่แพร่หลาย ในช่วงสองทศวรรษที่ผ่านมากระทรวงสาธารณสุขของประเทศไทย (MOPH) ร่วมกับศูนย์ควบคุมและป้องกันโรคของสหรัฐอเมริกา (CDC) ศึกษาภาระโรคไข้หวัดใหญ่ ต้นทุนและประโยชน์ของการฉีดวัคซีนไข้หวัดใหญ่ในประเทศไทย จากการที่ประเทศไทยมีระบบการรายงานโรคมายาวนาน กระทรวงสาธารณสุขได้จัดตั้งระบบเฝ้าระวังโรคปอดบวมและไข้หวัดใหญ่ขึ้น ระบบเฝ้าระวังนี้ให้ข้อมูลเชิงลึกเกี่ยวกับฤดูกาลการเกิดโรคและประชากรที่มีความเสี่ยงของโรครุนแรง การเกิดโรคไข้หวัดนกในคนในปี พ.ศ. 2547 กระตุ้นให้เกิดความกังวลต่อความเป็นไปได้ของการระบาดใหญ่ของไข้หวัดใหญ่ ความกังวลดังกล่าวร่วมกับข้อมูลความคุ้มค่าของการฉีดวัคซีนไข้หวัดใหญ่เร่งให้เกิดนโยบายการให้วัคซีน นโยบายของการเฝ้าระวังและการให้วัคซีนเพิ่มมากขึ้นจากช่วงการระบาดใหญ่ของ ไข้หวัดใหญ่ 2009 (H1N1) และ การระบาดของ COVID-19 บทความนี้เขียนจากการทบทวนข้อมูลในอดีตผ่านประสบการณ์ของประเทศไทยในการพัฒนานโยบายการเฝ้าระวังไข้หวัดใหญ่และการฉีดวัคซีนไข้หวัดใหญ่
